# Preliminary Investigation of the Process Capabilities of Hydroforging

**DOI:** 10.3390/ma9010040

**Published:** 2016-01-12

**Authors:** Bandar Alzahrani, Gracious Ngaile

**Affiliations:** Department of Mechanical and Aerospace Engineering, North Carolina State University, 911 Oval Drive-3160 EB3, Raleigh, NC 27695-7910, USA; baalzahr@ncsu.edu

**Keywords:** hybrid process, hydroforging, tube hydroforming process, lightweight

## Abstract

Hydroforging is a hybrid forming operation whereby a thick tube is formed to a desired geometry by combining forging and hydroforming principles. Through this process hollow structures with high strength-to-weight ratio can be produced for applications in power transmission systems and other structural components that demands high strength-to-weight ratio. In this process, a thick tube is deformed by pressurized fluid contained within the tube using a multi-purpose punch assembly, which is also used to feed tube material into the die cavity. Fluid pressure inside the thick tube is developed by volume change governed by the movement of the punch assembly. In contrast to the conventional tube hydroforming (THF), the hydroforging process presented in this study does not require external supply of pressurized fluid to the deforming tube. To investigate the capability of hydroforging process, an experimental setup was developed and used to hydroforge various geometries. These geometries included hollow flanged vessels, hexagonal flanged parts, and hollow bevel and spur gears.

## 1. Introduction

There is a growing demand for lightweight structures in the automotive, aerospace, and maritime industries. One of the ways to achieve significant weight reduction is exploring new and innovative manufacturing techniques. In metal forming, the combination of two or more processes is called hybrid forming process. Combining two or more forming processes have been used to develop metal forming processes thereby increasing the process productivity, enhancing part quality, increasing metal formability, reducing the overall process cost, and producing parts with features that would have not been feasible to produce. Attempts to hybridize metal forming processes have been done by several researchers; for example, Debin *et al.* [1] combined an isothermal closed forging and piercing process into a micro-scale hybrid forging process to produce a micro-double gear. A hybrid laser-assisted incremental sheet forming combined with stretch forming lead to reduction in cycle time and increase in formability [2]. A hybrid process combining warm and electromagnetic forming of magnesium alloy sheet has been reported [3]. Penda and Ngaile [4] introduced a new drawing process that incorporate attributes of hydroforming. Hybrid processes which combines deep drawing and cold forging, hot extrusion and integrated equal channel angular pressing, and a combination of tube spinning and a tube bending have been presented [5].

The tube hydroforming (THF) and forging processes are used widely in the industry. The THF process utilizes internal fluid pressure and restrictive die shape to form tubular shapes. In contrast with other many forming processes, THF uses a soft tool (fluid) to deform material, making it useful in producing hollow products with a high strength-to-weight ratio. However, the tube hydroforming process is limited to thin tubular components due to the load and power required to deform the tube [6,7]. Forging is a bulk metal forming process used to shape metal and increase its strength by hammering or pressing. Forging processes play a major role in the automotive industry where highly complex and dimensionally accurate parts are produced with enhanced material characteristics [8]. A large percentage of forged products for the automotive industry are used in power transmission units. Since forged products originate from solid billets, they are usually bulky. There is a great interest in reducing the weight of power transmission units by utilizing hollow structures. Combining unique features of THF and forging processes has the potential to open avenues for producing hollows structures that would have been difficult to produce if individual processes were to be used.

Hydroforging is a hybrid metal forming process which combines forging and hydroforming operations. The unique characteristics emanating from forging and hydroforming processes, make this hybrid process ideal for manufacturing of thick walled tubular components. In the last decade, extensive research work on a similar variant of hydroforging process was carried out at the University of Strathclyde in the UK [9,10,11,12,13,14,15,16]. The process was referred to as injection forging. In this process, a pressurized polymeric material is inserted inside a thick tube while a movable punch is used to feed the tube and pressurize the polymeric material. Different types of polymeric materials were studied and design guidelines for the process were outlined. While the use of polymeric materials as pressure medium was easier to implement, establishment of optimal pressure loading for a specific part is not feasible. Furthermore, the process require secondary operation to remove the injected material. Experimental investigations of hydroforging where fluid is used as a pressure medium were carried out to produce simple bulge shapes by several researchers [17,18]. The aim of this paper is to present experimental investigation on the process capabilities of hydroforging. The investigation was focused on hollow flanged vessels, hexagonal flanged parts, and hollow bevel and spur gears.

## 2. Hydroforging Process Window and Design Aspects

**Figure 1:**
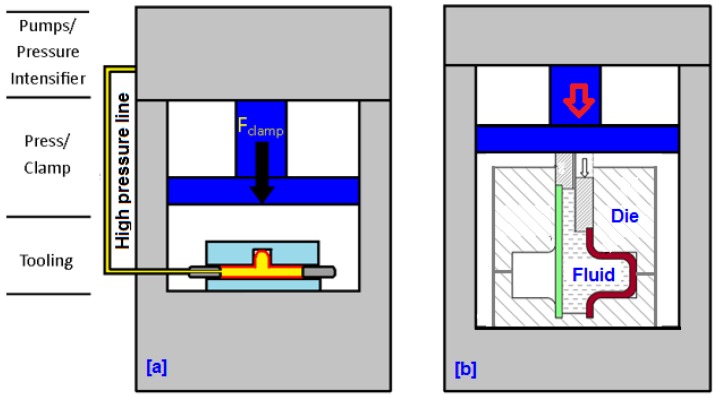
(**a**) Conventional THF; (**b**) Tube hydroforging process.

In order to provide a clear distinction between hydroforging and the conventional tube hydroforming, we first highlight the main characteristics of the two processes. Figure 1a shows a schematic diagram of a conventional tube hydroforming (THF). The major components of a THF process are the press, of which the function is to close the die set, and the pressure intensifier that is used to supply high pressure fluid to the tube. One of the limitations of THF is that it is difficult and cost prohibitive to hydroform thick tubes as the THF system would require very large pressure intensifier. A schematic diagram of the hydroforging process is given in Figure 1b. In this process, a thick tube is deformed by pressurized fluid contained within the tube during the upsetting process. A punch assembly is used to feed tube material into the die cavity. The pressure is generated by compressing the fluid volume contained within the tube. During deformation, no fluid is supplied to the deforming specimen. Principally, the pressure created by pushing the fluid should be greater than the required pressure to form the part without defects. To achieve the desired load path, a pressure relief valve is used to adjust the pressure by releasing fluid. The control architecture for this process is such that at time, *t*, the fluid pressure generated in the chamber is communicated to the data acquisition center. We envision that the architecture of a hydroforging press to be used in an industrial setting will be similar to conventional forging presses with few additional auxiliary units, namely punch assembly and fluid pressure control units.

**Figure 2:**
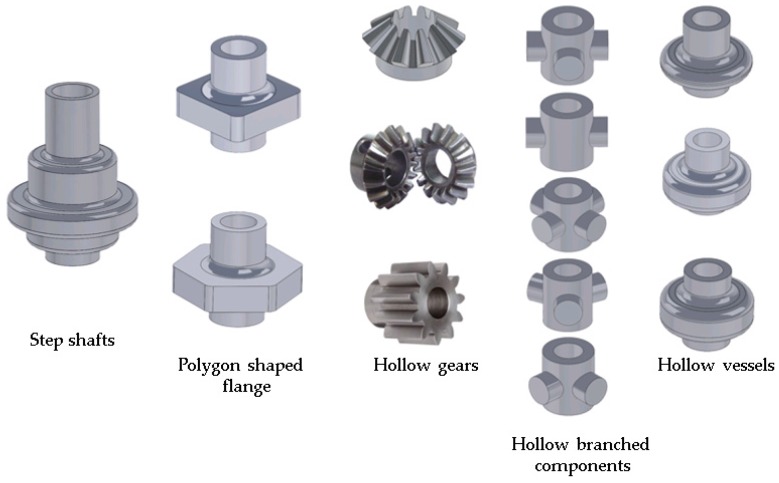
Families of potential candidate parts for hydroforging.

There is a potential to produce different types of products with high strength-to-weight ratio using hydroforging process. Figure 2 shows families of potential candidates for this process, which includes step shafts, polygonal shaped flanges, hollow gears, hollow branched components and hollow vessels.

**Figure 3:**
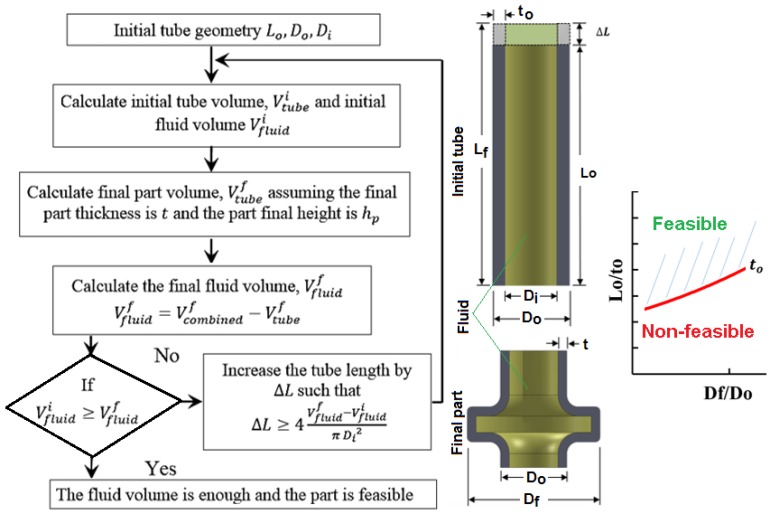
Volume calculation scheme.

Since in hydroforging no external fluid is supplied to the tube, the process window for hydroforging is thus largely dependent on the initial fluid volume in the tube. A part would be feasible to form if the initial fluid volume is enough to generate the required pressure throughout the process. To investigate the potential candidates for this process, volume calculations for both the tube and the fluid at the initial and final forming stages were performed for several geometries. Flow diagram for volume calculation is given in Figure 3. The main output of the volume calculations was the tube length suitable to produce a product by the hydroforging process. For a specific part geometry, a process window can be built by plotting the flange to tube outer diameter ratio$\;D_{f}/D_{o}$ *versus* the initial tube length to thickness ratio$\;L_{o}/t_{o}$ at several tube thicknesses. The generated curves provide process limit, *i.e.*, the regions below the curves represent non-feasible region while above the curves signify feasible regions.

### 2.1. Process Window for Hollow Vessels

**Figure 4:**
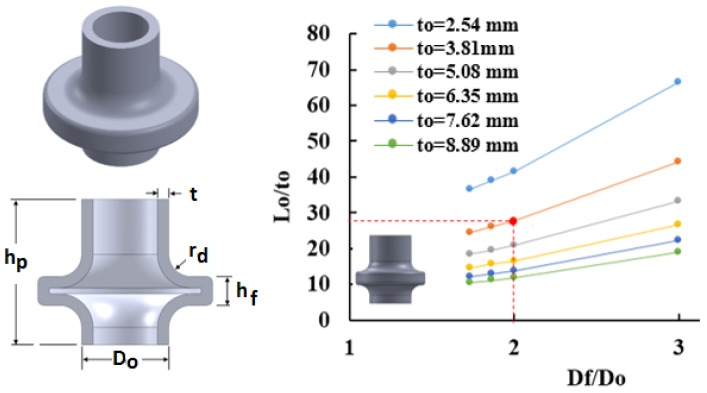
Process window for hollow vessel with flange height *h_f_* = 12.7 mm.

Process windows were constructed for hollow vessels using three flange heights: $h_{f}=12.7\text{ mm}$, $h_{f}=19.05\text{ mm}$, and $h_{f}=25.4\text{ mm}$. Figure 4 shows a process window for hollow vessels with a flange height of $h_{f}=12.7\text{ mm}$ at six different tube thicknesses (2.54 mm, 3.81 mm, 5.08 mm, 6.35 mm, 7.62 mm, and 8.89 mm). For a selected tube thickness, the part is considered to be feasible above the line and infeasible under the line. For instance, a hollow vessel with a flange to tube outer diameter ratio$\;D_{f}/D_{o}$ of 2 is feasible to form using a 3.81 mm thick tube if the tube length $L_{o}\geq 105.5\text{ mm}$ as shown in Figure 4. A shorter tube will not provide enough fluid to generate the required pressure to form the part.

### 2.2. Process Window for Hollow Hexagon Flanges

**Figure 5:**
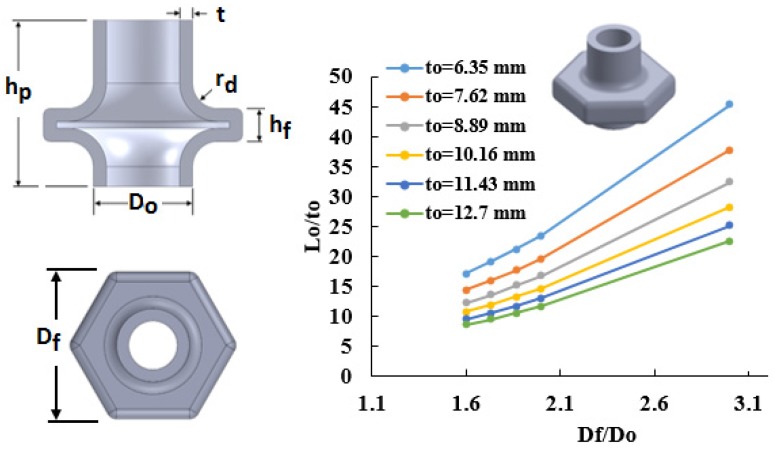
Process window for hollow hexagonal with flange height *h_f_* = 25.4 mm.

Process windows were constructed for hollow hexagon shaped flanges using three flange heights, $h_{f}=12.7\text{ mm}$, $h_{f}=19.05\text{ mm}$, and $h_{f}=25.4\text{ mm}$. Figure 5 shows a constructed process window for a flange height of $h_{f}=25.4\text{ mm}$ at six different tube thicknesses (6.35 mm, 7.62 mm, 8.89 mm, 10.16 mm, 11.43 mm and 12.7 mm). For example, a hollow hexagon shaped flange with a flange to tube outer diameter ratio$\;D_{f}/D_{o}$ of 3 is feasible to form using a 12.7 mm thick tube if the tube length $L_{o}\geq 266\text{ mm}$. The flange diameter is defined as the distance between two parallel flange surfaces.

### 2.3. Process Window for Hollow Gears

**Figure 6:**
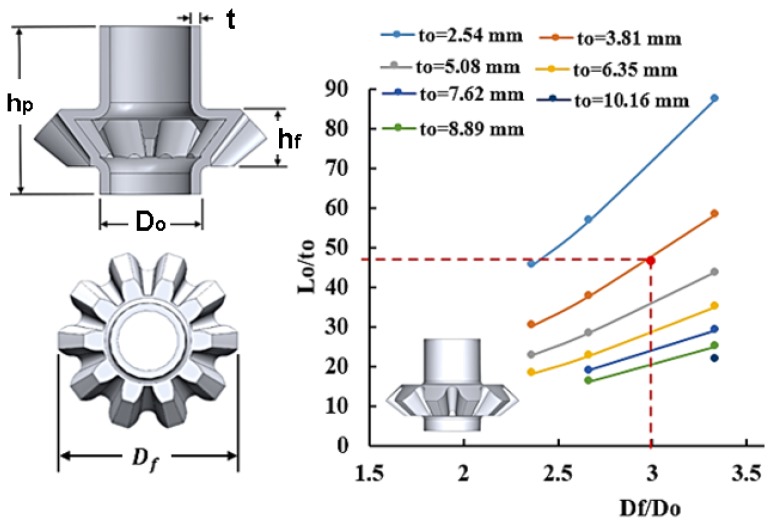
Process window for a ten teeth hollow bevel gear.

**Figure 7:**
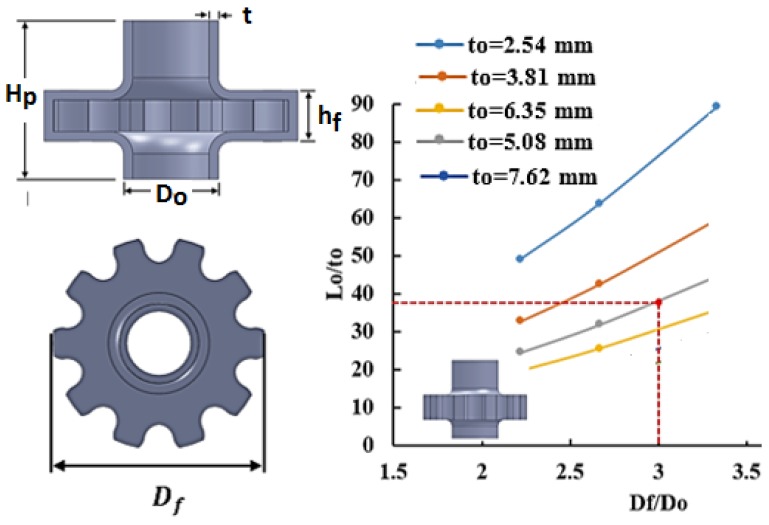
Process window for a ten teeth hollow spur gear.

For a ten teeth hollow bevel gear, a process window for a flange height of $h_{f}=21.59\text{ mm}$ was constructed using seven tube thicknesses (2.54 mm, 3.81 mm, 5.08 mm, 6.35 mm, 7.62 mm, 8.89 mm, and 10.16 mm) as shown in Figure 6. For instance, a hollow bevel gear with a flange to tube outer diameter ratio$\;D_{f}/D_{o}$ of 3 is feasible to form using a 3.81 mm thick tube if the tube length $L_{o}\geq 183\text{ mm}$. Similarly, a process window was constructed for hollow spur gear with a flange height of $h_{f}=19.05\text{ mm}$ at six different tube thicknesses (2.54 mm, 3.81 mm, 5.08 mm, 6.35 mm, 7.62 mm, 8.89 mm, and 10.16 mm) as shown in Figure 7. With this example, a hollow spur gear with a flange to tube outer diameter ratio$\;D_{f}/D_{o}$ of 3 is feasible to form using a 5.08 mm thick tube if the tube length $L_{o}\geq 190.5\text{ mm}$.

**Figure 8:**
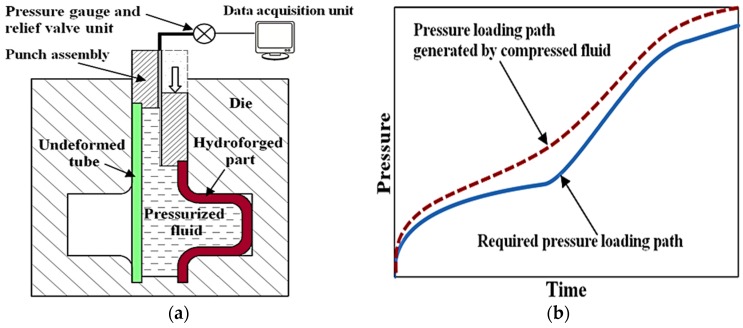
Hydroforging (**a**) and pressure loading schematic (**b**).

It should be noted that the fluids used in hydroforging will be compressible, thus, slightly longer tube will be needed to account for the compressibility factor. Furthermore, control of the optimal pressure loading path may require release of fluid from the tube. Figure 8 shows conceptual two pressure profiles for a hydroforging process one representing an optimal pressure required to successfully form the part and the other profile represent induced fluid pressure due to upsetting of the tube. The control should be such that at time, *t*, some fluid is released to match the desired pressure. This implies that longer tubes beyond that shown in the process windows will be needed. These constraints indicate that hydroforging process will be practical and cost effective only for certain family of parts. One of the major benefits, however, is that very high pressure sufficient to hydroforge thick tubing can be generated during upsetting of the tube.

## 3. Experimental Setup Development

**Figure 9:**
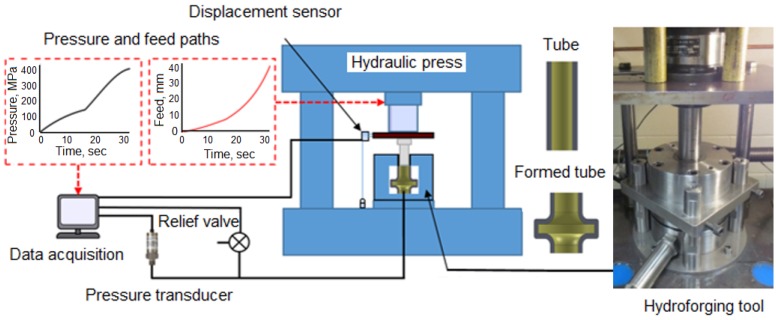
Schematic of hydroforging experimental setup.

A schematic of the hydroforging setup is shown in Figure 9. The setup consists of a 150 ton hydraulic press that houses the tooling assembly. The tooling assembly is connected to the pressure lines, relief valve, pressure transducer, and data acquisition system. The hydroforging tooling assembly consists of die inserts, die housing, multi-purpose punch assembly, tube blank, and a sealing insert.

**Figure 10:**
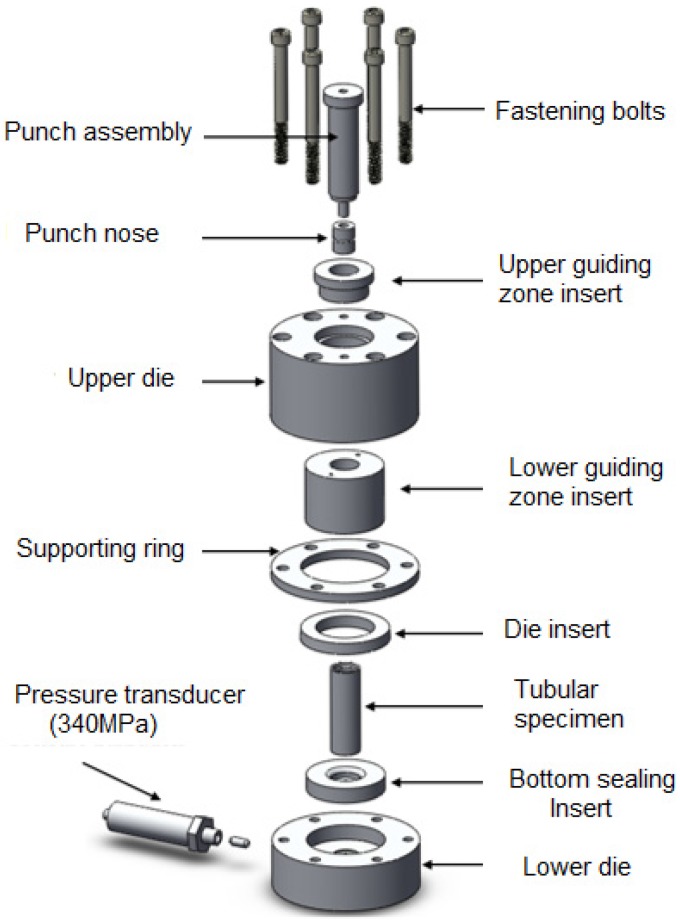
Exploded model for tube hydroforging tooling.

**Figure 11:**
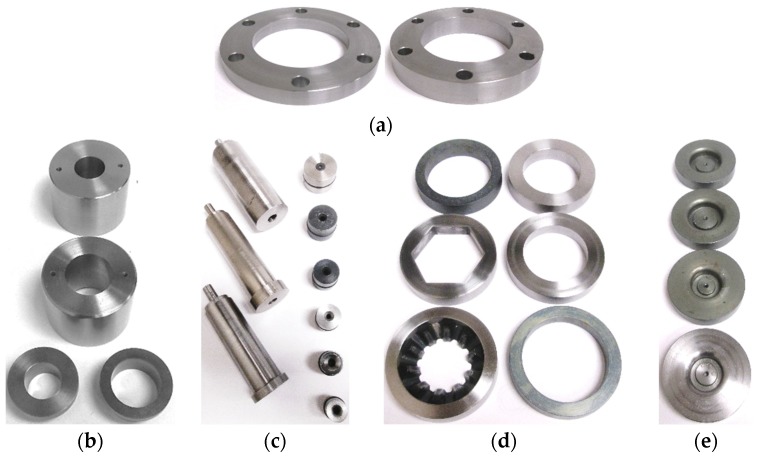
Experimental setup parts: (**a**) Support rings; (**b**) guiding zone inserts; (**c**) punch assemblies; (**d**) die inserts; and (**e**) bottom sealing inserts.

The tube hydroforging tooling was designed with interchangeable die inserts so that a variety of tubular materials and geometries could be tested. An exploded view of the hydroforging tooling is shown in Figure 10. The figure shows lower die housing, upper die housing, bottom sealing insert, die insert, supporting ring, guiding zone inserts, punch assembly fastening bolts, tube sample, and a pressure transducer. The punch assembly consists of the punch body and the punch nose. The upper die housing, the lower die housing, and supporting ring are designed to be universal parts. These parts were fabricated to meet the requirement of several tube sizes up to 50.8 mm OD, flange die cavity up to 88.9 mm OD, and flange height of up to 25.4 mm. The punch nose, the guiding zone inserts, the bottom sealing inserts, and the die inserts were designed to meet the required tube sizes and thicknesses. The punch nose and guiding zone inserts were made out of A2 tool steel and hardened to 59 HRC to be able to handle high pressure loads. Punch bodies were fabricated from alloy steel for each tube size. Figure 11 shows a set of fabricated parts made to customize the tube sizes, tube thicknesses, and flange heights.

### Experimental Matrix and Test Procedures

**Figure 12:**
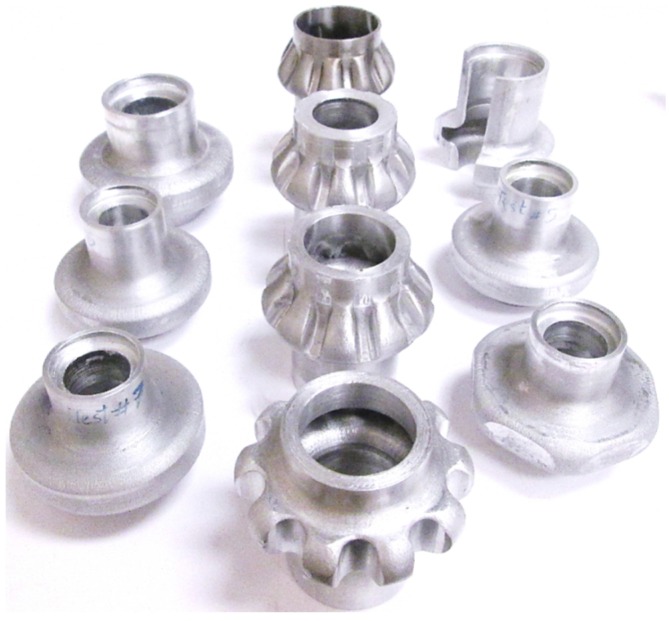
Hydroforged parts.

Aluminum (AL6061) and stainless steel (SS 304) tube samples were used to perform the experiments. The properties for these materials were as follows; for AL6061 the strength coefficient *K* = 118 MPa and strain hardening exponent *n* = 0.22. The strength coefficient and strain hardening exponent for SS304 were 1426 MPa and 0.5, respectively. The experimental matrix is given in Table 1. In the table, the tube dimensions are described by the tube outer diameter $D_{o}$, the tube thickness $t_{o}$, and the tube length, $L_{o}$. The die dimensions are defined by the flange height$\;h_{f}$, and the flange diameter $D_{f}$. Annealed AL6061 and annealed SS304 tubes were used to perform the experiments. Before the experiments all the tubular specimens were wrapped with Teflon sheet that acted as a lubricant. The tube was filled with oil and the punch is pre-assembled to establish contact with the tube end. ISO 32 light hydraulic oil with a viscosity of 32 centistoke was used. The punch is then pushed downward by a 150 ton press. As the tube is pushed towards the cavity, fluid pressure is generated inside the tube due to volume change, forcing the material to flow toward to die cavity wall. During the process, fluid pressure and punch stroke are recorded as a function of time. The pressure control system is installed with 350 MPa pressure transducer. Figure 12 shows a variety of parts that were hydroforged which included hollow vessels, hollow hexagonal shaped flanges, and hollow spur and bevel gears.

**Table 1:** Experimental matrix.

Tube Dimensions and Material				Die Dimensions		Hydroforged Parts
$D_{o},\;mm$	$t_{o},mm$	$L_{o},mm$	Material	$h_{f},mm$	$D_{f},mm$	
38.1	6.35	127	AL6061 annealed	18.5	76.2	Hollow vessel-6.35 mm thick
38.1	6.35	127	AL6061 annealed	21.6	76.2	Hollow vessel-21.6 mm flange
38.1	8	127	AL6061 annealed	18.5	76.2	Hollow vessel-8 mm thick
38.1	6.35	127	AL6061 annealed	17.8	76.2	Hollow hexagon flange
50.8	6.35	114	AL6061 annealed	18.5	76.2	Hollow vessel-6.35 mm thick
50.8	9.5	120	AL6061 annealed	18.5	88.9	Hollow vessel-9.5 mm thick
50.8	6.35	101	AL6061 annealed	20.3	76.2	Hollow spur gear-AL6061
50.8	9.5	108	AL6061 annealed	17.8	76.2	Hollow bevel gear-Al6061
50.8	1.65	114	SS304 annealed	17.8	76.2	Hollow bevel gear-SS304

## 4. Experimental Results and Discussion

**Figure 13:**
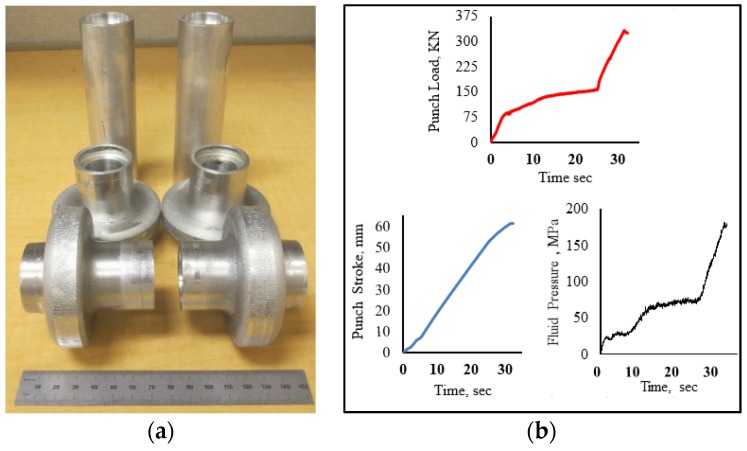
(**a**) Hollow vessel, flange = 18 mm and 21 mm; (**b**) loading for 18 mm flange.

**Figure 14:**
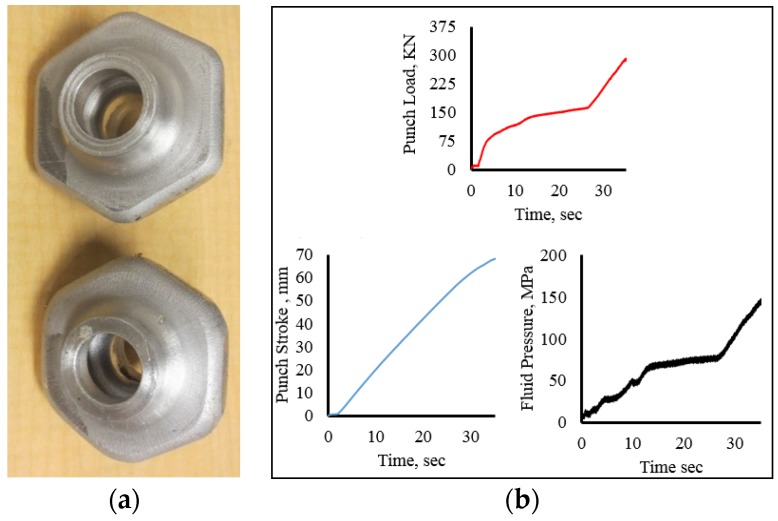
(**a**) Hexagonal hollow parts; (**b**) loading paths.

Figure 13a shows hydroforged hollow vessel with flange sizes of 18 mm and 21 mm. These parts were hydroforged from 38 mm OD × 127 mm long tube with a wall thickness of 6.35 mm. The loading paths that resulted from the hydroforging operations are given in Figure 13b. The experiments were carried out in 35 s and attained a maximum punch force of 325 kN and maximum fluid pressure of 180 MPa. To form this part a feed of 60 mm was used. Figure 14 shows hydroforged hexagonal shaped hollow parts with a flange thickness of 18 mm and the corresponding loading paths. These parts were hydroforged using 38 mm OD × 127 mm long tubular samples with a wall thickness of 6.35 mm. The maximum punch load and fluid pressure of 300 kN and 160 MPa, respectively, were attained at maximum punch stroke of 68 mm.

**Figure 15:**
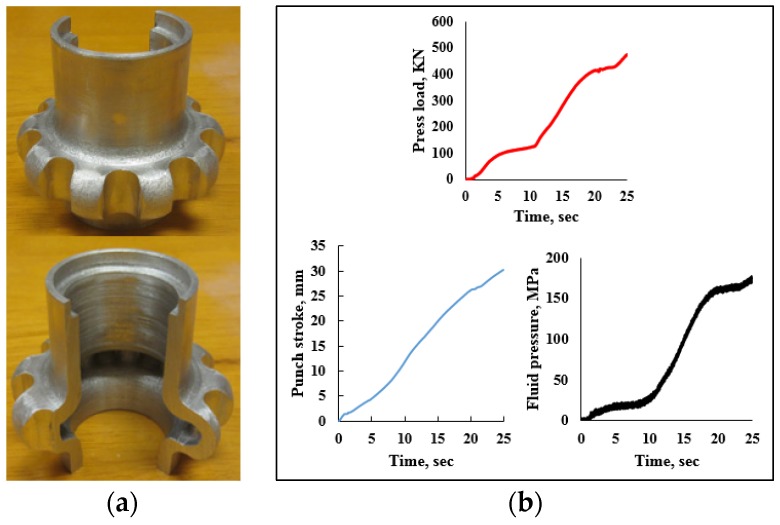
(**a**) Spur gear; (**b**) loading paths.

**Figure 16:**
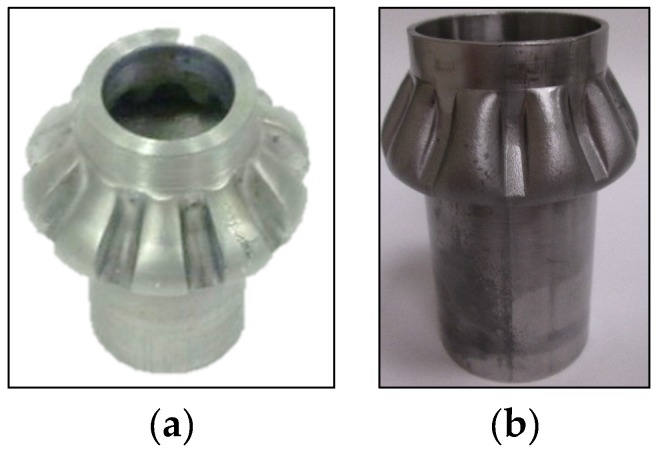
Bevel gear: AL6061(**a**) and SS304 (**b**).

A ten teeth spur gear was hydroforged using a 50.8 mm OD × 6.35 mm aluminum tubes as shown in Figure 15. The corresponding loading paths are given in Figure 15b. The maximum press load reached 474 kN at the end of the process. During teeth forming, the press load increased from 130 kN to 474 kN and a maximum fluid pressure of 175 MPa was reached at punch stroke of 30 mm. In this test, the spur gear was not fully formed. The tube material at the tooth root was subjected to excessing shearing causing rupture. Hydroforging of bevel gears from AL6061 and SS304 was also conducted, as shown in Figure 16.

**Figure 17:**
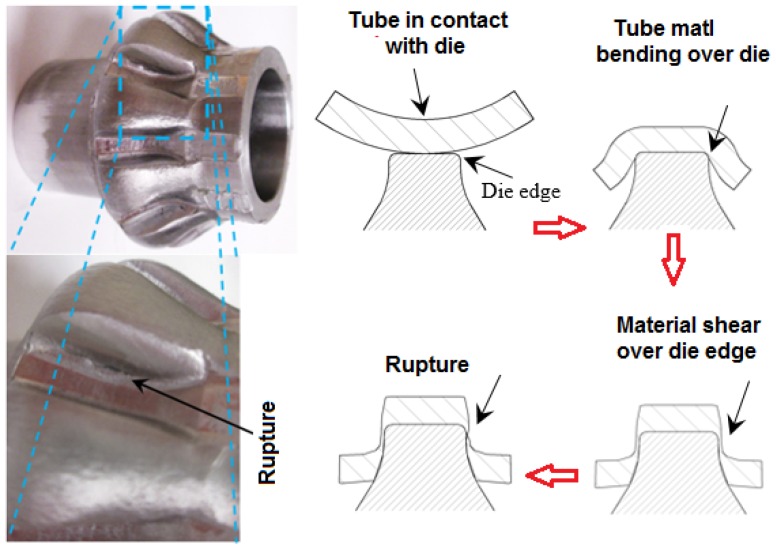
Material failure mode during hydroforging of a bevel gear from AL6061 blank.

Part failures were observed in several parts formed by the tube hydroforging process. The failure modes in these parts can be classified into three categories: tube rupture, surface cracks and excessive thinning. Figure 17 shows the experimental evidence of tube rupture in a bevel gear formed using a 6.35 mm thick aluminum tube. In this experiment, the tubular material was pushed by the generated pressure against the die teeth causing excessive shear as the tooth was being formed. The failure progression is illustrated in Figure 17. The tube material was exposed to shearing at the sharp die teeth edges leading to rupture. To reduce the shear effect, an extra forming stage may be needed with larger die radius. When a thicker aluminum tube (*t*_o_ = 9.5 mm) was used to form the bevel gear, no rupture was observed.

**Figure 18:**
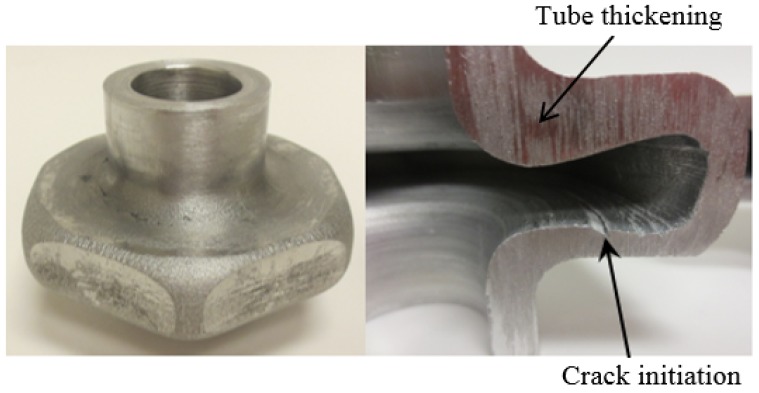
Crack initiation and non-uniform thickness distribution on hexagonal shaped flange.

Few samples exhibited internal surface cracks at the bottom side of a hexagon shaped flange shown in Figure 18. The crack initiated during the expansion stage where the tube undergo tensile loading caused by excessive thinning at this location. Tube thickening can be observed at the top portion of the flange. This failure would be avoided if more material was fed inside the die cavity in advance of pressure. It should be noted that the pressure loading paths used in this study were not optimized. However, they were sufficient to demonstrate the feasibility of the hydroforging process. The above mentioned failures could be avoided by controlling the generated pressure in which more material is fed into the die cavity and subsequently delaying or completely eliminating crack initiation and tube thickening. Future work will include determination and control of optimal pressure loading that lead to uniform wall thickness distribution.

## 5. Conclusions

Investigation on the feasibility of hydroforging process where thick tubes are formed by combining forging and hydroforming operations was carried out. Based on initial fluid volume responsible for inducing pressure inside the tube during upsetting of the tube, process windows for determining the required tube length to successfully form a part were established for a few geometries. Preliminary hydroforging experiments were carried out to examine the feasibility and limitations of this process. Hollow flanged vessels, hollow flanged hexagonal parts, and hollow bevel and spur gears were hydroforged from AL6061 and SS306 tubular blanks. The conclusions drawn from this preliminary study are:

- Hydroforging process has a lot of potential for manufacturing hollow components with high-strength-to-weight ratio from thick tubing for applications in power transmission systems.
- The simplicity of the hydroforging tool set up implies that development of hydroforging production machines may not be capital intensive compared to conventional hydroforming machines. This is largely due to the fact that hydroforging does not require a pressure intensifier. Thus, a conventional forging machine could be modified to suit the requirements of the hydroforging process.
- One of the drawbacks of hydroforging process, is that the induced pressure inside the tube relies on the initial volume of fluid in the tube. Thus, the hydroforging process is only applicable for products whose internal tube volume continuously decrease as the tube material is fed toward the die cavity.
- Since the control of fluid pressure for ensuring that the pressure loading path is optimal may be achieved by releasing some of the pressurized fluid inside the tube, much longer tubular blanks to compensate for the release of fluid will be needed.
